# Fractionation of dicarboxylic acids produced by *Rhizopus oryzae* using reactive extraction

**DOI:** 10.1038/s41598-022-06069-y

**Published:** 2022-02-07

**Authors:** Lenuta Kloetzer, Alexandra Tucaliuc, Anca-Irina Galaction, Dan Caşcaval

**Affiliations:** 1 “Cristofor Simionescu” Faculty of Chemical Engineering and Environmental Protection, “Gheorghe Asachi” Technical University, D. Mangeron 73, 700050 Iasi, Romania; 2 Faculty of Medical Bioengineering, “Grigore T. Popa” University of Medicine and Pharmacy, M. Kogalniceanu 9-13, 700454 Iasi, Romania

**Keywords:** Biotechnology, Chemistry, Engineering

## Abstract

Fumaric, malic, and succinic acids have been selectively separated from their mixture obtained by *Rhizopus oryzae* fermentation using reactive extraction with Amberlite LA-2 dissolved in three solvents with different dielectric constants (n-heptane, n-butyl acetate, and dichloromethane). This technique allows recovering preferentially fumaric acid from the mixture, the raffinate containing only malic and succinic acids. The extractant concentration and organic phase polarity control the efficiency and selectivity of acids extraction. The increase of aqueous phase viscosity reduces the extraction yield for all studied acids, but exhibits a positively effect on separation selectivity. By using Amberlite LA-2 concentration equal to that stoichiometrically required for interfacial reaction with fumaric acid and mixing intensity which does not allow higher diffusion rates for larger molecules (malic and succinic acids), the maximum value of fumaric acid extraction rate exceeds 90%, while the selectivity factor value becomes 20. Regardless of the extraction system, the complete separation of fumaric acid from their mixture is possible by multi-stage extraction process, adjusting the extractant concentration in each stage. At higher values of aqueous phase viscosity, more extraction stages are required, while the increase of solvent polarity reduce the required number of stages for total recovery of fumaric acid.

## Introduction

Due to its nontoxic behaviour, fumaric acid has various current applications in polymer industry, as starting material for polymerization and esterification reactions, in food industry, as additive for preservation, flavouring, coagulation, and acid regulation^1^. Fumaric acid can be produced chemically from maleic anhydride, benzene or n-butane, as well as by fungal fermentation processes using especially *Rhizopus oryzae* and *Rhizopus arrhizus*^2–5^. The disadvantage related to the chemical technologies (high materials and energy consumption, raw materials cost fluctuation) has increased the interest in the fumaric acid production by fermentation. Biosynthesis process by filamentous fungi of *Rhizopus oryzae* offers the main advantage of eco-friendly solutions, the use of low-cost substrates and satisfactory yields related to the raw materials.

Besides the increased interest for fermentation process, the downstream processes raise important problems concerning the environmental protection^5^. *Rhizopus oryzae* fermentation processes leads to the accumulation into the broth of a mixture of carboxylic acids, containing fumaric acid as the main product and small amounts of by-products, such as malic and succinic acids^6,7^. Generally, acids mixture includes: 85–90% fumaric acid, 7–9% malic acid, and 2–7% succinic acid^6,7^.

The most applied techniques for separating these acids at laboratory or industrial scale consist on electrodialysis, crystallization, ion exchange absorption, and precipitation as calcium salt^4^. However, the major inconvenient of these methods is represented by the generation of waste products, mainly solid wastes of calcium sulphate sludge and acidic wastewaters^4^.

Among the widely used separation methods, liquid–liquid extraction represents a viable technology in terms of accessibility and efficiency. However, extraction has limited applications on carboxylic acids separation, due to their low solubility in organic solvents. In this context, the physical extraction of fumaric acid with organic solvents occurs with low yields^8^. Also, for secondary acids, particularly malic and succinic acids, the maximum extraction yields of approx. 30% are reached by using long chain aliphatic alcohols^9^.

The performance of extraction process can be enhanced by reactive extraction with an extractant added into the organic phase. The previous studies on individual separation of fumaric, malic, and succinic acids by reactive extraction with Amberlite LA-2 or tri-n-octylamine indicated that this method could be an efficient alternative for the currently applied techniques^8–10^. According to these experiments, the reactive extraction yield reached over 95% depending mainly on the polarity of organic phase.

In this regard, the current experiments investigate the selective separation of the acids mixture (fumaric, malic, and succinic acids) obtained by fungal fermentation with *Rhizopus oryzae* using reactive extraction with Amberlite LA-2. Because the solvent polarity controls the extraction efficiency and selectivity, the experiments have been carried out using three solvents whose dielectric constants cover a large range of values (dichloromethane, n-butyl acetate, n-heptane). Therefore, the separation performance and, implicitly, selectivity have been analysed in direct correlation with the polarity of the considered solvents.

## Results and discussion

Previous experiments on the individual reactive extraction with an amine extractant of these three dicarboxylic acids, namely fumaric, malic and succinic acids, led to the assumption that the separation is achieved by an interfacial reaction between solute and extractant, the structure of the extracted compound depending mainly on the organic phase polarity^8,10^. The interfacial reaction that describes the reactive extractive process respects the general mechanism given in Eq. (1):

1 $$ {\text{m R}}\left( {{\text{COOH}}} \right)_{{{2}({\text{aq}})}} + {\text{ p Q}}_{{({\text{o}})}} \rightleftarrows \left[ {{\text{R}}\left( {{\text{COOH}}} \right)_{{2}} } \right]_{{\text{m}}} \cdot {\text{Q}}_{{{\text{p}}({\text{o}})}} $$

(Q symbolizes the aminic extractant).

The dielectric constant of the organic solvents controls the formation of amine type adducts by hydrogen bonds (p > 1). The number of extractant molecules included in the structure of the interfacial complex decreases by increasing polarity of the organic phase or by ionization of the carboxylic group^8,10^. This phenomenon leads to the decrease of the extraction constant. However, if the mechanism of the interfacial reaction is not changed by varying the dielectric constant value, the increase of the solvent polarity induces a positive effect on the extraction constant, due to the improvement of the solubilisation capacity of the interfacial complex by the organic phase^8,10^^.^

**Figure 1:**
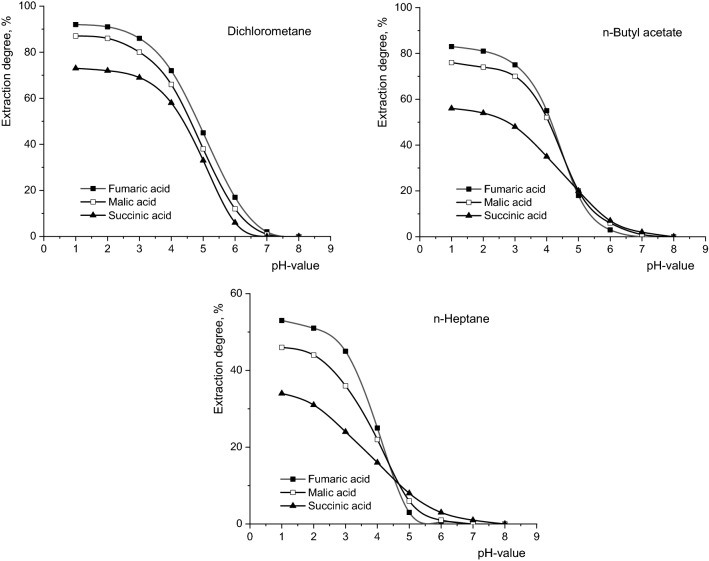
Influence of pH-value on reactive extraction efficiency of fumaric, malic, and succinic acids (Amberlite LA-2 concentration = 50 g/l, mixing intensity = 0.25 m/s, aqueous phase viscosity = 1 cP).

Previous studies are continued and developed by investigating the selective separation of fumaric, malic and succinic acids from the biosynthetic mixture using the reactive extraction with Amberlite LA-2, under conditions of various polarities of the organic phase. The pH value of the aqueous phase controls the efficiency of the reactive extraction, regardless of the type of dicarboxylic acid or of the polarity of the organic phase. For all the studied acids the increase of the pH value leads to the reduction of the reactive extraction efficiency (Fig. 1). This phenomenon is more pronounced for pH values higher than 3 for fumaric and malic acids, respectively higher than 4 for succinic acid. This gap is the result of the partial dissociation of these acids, at a single carboxyl group, processes corresponding to different values of pK_a1_ (at 25 °C, pK_a1_ = 3.03 for fumaric acid, pK_a1_ = 3.40 for malic acid, pK_a1_ = 4.16 for succinic acid ^13^).

Obviously, the increase of the acidity of a compound facilitates the reaction with amine extractant and, implicitly, increases the efficiency of the reactive extraction. Therefore, in the case of all used organic solvents, for pH values below 4, the extraction order was fumaric acid > malic acid > succinic acid. Above this pH value, the dielectric constant of the solvent controls the separation efficiency. Thus, as can be seen from Fig. 1, for dichloromethane the order of extraction mentioned above is maintained for the entire experimental range of pH values. But, if the extraction is performed in solvents with lower polarity, for pH values above 4, the extraction efficiency decreases in a reverse order: succinic acid > malic acid > fumaric acid, this phenomenon being more obvious for n-heptane.

The extraction sequence for pH > 5 is similar to the increase sequence of pK_a2_ values (at 25 °C, pK_a2_ = 4.44 for fumaric acid, pK_a2_ = 5.20 for malic acid, pK_a2_ = 5.60 for succinic acid^11^). Due to the lower solubilisation capacity of ionized compounds by solvents with lower polarity, for pH values higher than 4, n-butyl acetate and n-heptane will solubilize the dicarboxylic acids with lower dissociation degree of carboxylic groups, namely succinic and malic acids, whose pK_a2_ being higher than 5.

**Figure 2:**
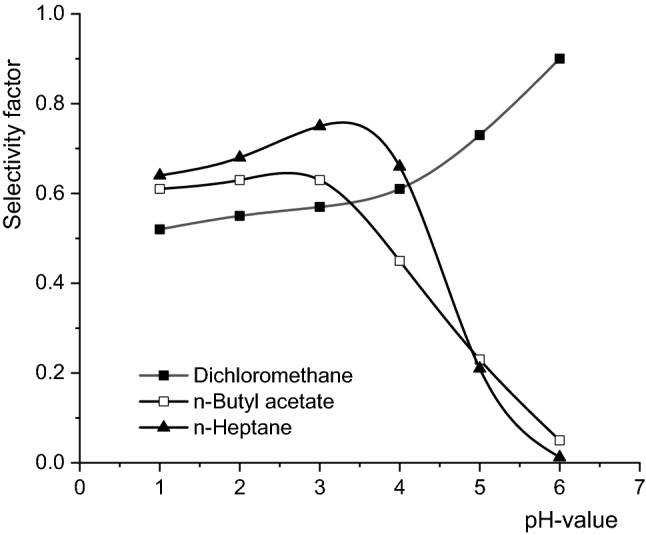
Influence of pH-value on selectivity factor (Amberlite LA-2 concentration = 50 g/l, mixing intensity = 0.25 m/s, aqueous phase viscosity = 1 cP).

A more suggestive analysis of the effects of solute acidity and solvent polarity on the efficiency of selective separation can be achieved by establishing the influence of the pH value of the aqueous phase on the selectivity factor, defined as the ratio between the extraction yield of fumaric acid and the sum of extraction yields of malic and succinic acids. According to Fig. 2, the dependence between the selectivity factor and the pH is controlled by the polarity of the solvent. Thus, there are important differences between the variation recorded for dichloromethane and those for n-butyl acetate and n-heptane.

In all the extraction systems analysed, the selectivity factor increases slightly by increasing the pH value to 4 of, as the result of the superior acidity of fumaric acid, which attenuates the effect of its partial dissociation. This tendency is maintained in the case of dichloromethane, a solvent with superior capacity to solubilize ionized molecules, and becomes more pronounced for the pH values higher than 4, leading to the increase of the selectivity factor. Instead, in the extraction systems with organic solvents possessing low capacity to solubilize ionized molecules, n-butyl acetate and n-heptane, at pH values higher than 3, the selectivity factor strongly decreased. This behaviour is the result of the more efficient extraction of acids with a lower degree of dissociation at the second carboxylic group in the 3 to 6 pH-domain.

Figure 2 also indicates that in most cases the selectivity factor has values lower than 1, which suggests that pH is not a factor controlling the selectivity of reactive extraction. Values higher than 1 for the selectivity factor were recorded only for the extraction in dichloromethane and at pH value higher than 6, but these conditions are practically inefficient due to the very low yield of fumaric acid extraction.

The dependence between the extraction degree and the concentration of the extractant of amine type has been analysed by considering the influence of acidity on the rate of competitive reactions between the extracted dicarboxylic acids and Amberlite LA-2 and, implicitly, in the order of these acids extraction.

**Figure 3:**
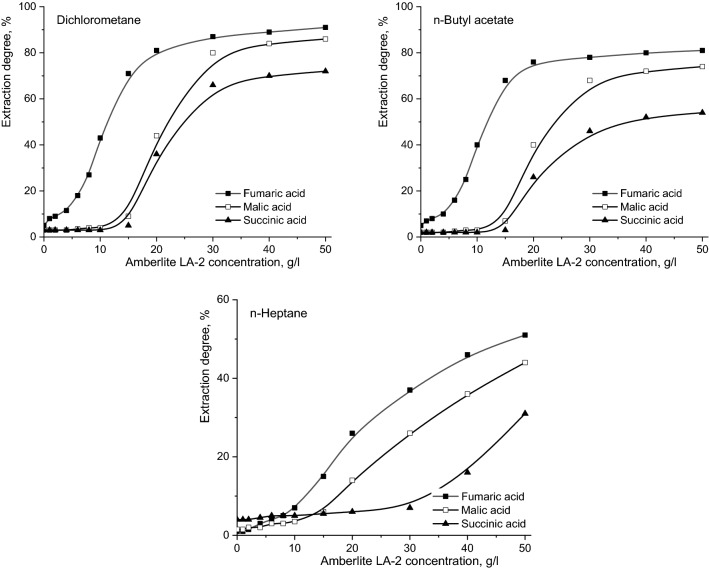
Influence of Amberlite LA-2 concentration on reactive extraction efficiency of fumaric, malic, and succinic acids (pH 2, mixing intensity = 0.25 m/s, aqueous phase viscosity = 1 cP).

The positive influence on the reactive extraction efficiency of increasing Amberlite LA-2 concentration in all the systems analysed is represented in Fig. 3. This effect represents a consequence of the increasing the amount of one reactant in the reaction region, respectively at the interfacial region. In addition, the influence of Amberlite LA-2 concentration in the extraction process of these dicarboxylic acids can be related to order of the acids extraction. Thus, at low extractant concentrations, it was found that only fumaric acid is extracted, its extraction yield increasing strongly with amine concentration increase to a certain value of its concentration, this effect becoming more attenuated for higher amine concentration. Practically, the change in the magnitude of this influence occurs around the value of Amberlite LA-2 concentration stoichiometrically needed for the interfacial reaction with fumaric acid. The value of the extractant concentration required for the fumaric acid reaction, according to the interfacial reaction mechanism, is 16 g/l for dichloromethane and n-butyl acetate, respectively 32 g/l for n-heptane^8^. For this reason, the transition in the variation slope of fumaric acid extraction degree with the extractant concentration occurs at 20 g/l Amberlite LA-2 for solvents with higher polarity and becomes insignificant for n-heptane, in the range of amine concentrations considered in the experiments. The increase of this concentration threshold is due to the diffusion limitations, as well as to the possibility of formation of fumaric acid molecules associations through intermolecular hydrogen bonds, which block the carboxylic groups and require higher amounts of extractant to make the interfacial reaction possible^12^.

Increasing the amount of Amberlite LA-2 in the interfacial region increases its ability to compete with the formed hydrogen bonds, respectively to break them, and, implicitly, to react with the carboxylic groups, leading to the improvement of the reactive extraction efficiency. The phenomenon of hydrogen bond formation is intensified by reducing the organic phase polarity. Therefore, variation of fumaric acid extraction yield remains rather constant for extractant concentration domain up to 50 g/l.

The compounds with lower acidity than fumaric acid are being reactively extracted at higher aminic extractant values than that corresponding to the stoichiometric requirement for the reaction with fumaric acid. Thus, according to Fig. 3, the extraction efficiency of malic acid begins to increase significantly for Amberlite LA-2 concentration values above 15–20 g/l, while for succinic acid for extractant concentration values higher than the extractant concentrations related to its stoichiometry needed for reacting with the first two dicarboxylic acids, respectively 25–30 g/l (the stoichiometric requirement for the reaction of Amberlite LA-2 with malic acid is 1.4 g/l for dichloromethane and n-butyl acetate, 2.8 g/l for n-heptane)^10^. Until the stoichiometric concentration of the extractant is reached, the compounds with lower acidity are predominantly extracted by physical processes, without a competitive co-extraction of malic and succinic acids with fumaric acid, respectively of succinic acid with fumaric and malic acids (Fig. 3).

Generally, the previous approaches are valid for all the studied extraction systems. The most pronounced effect of the Amberlite LA-2 concentration on the extraction efficiency were observed for solvents with higher dielectric constants, namely dichloromethane and n-butyl acetate. In these cases, the mechanisms of the interfacial reactions indicate the formation of compounds structure R(COOH)_2_.Q for fumaric and malic acids and R(COOH)_2_.Q_2_ for succinic acid, thus justifying the mentioned values of the extractant concentration necessary for the stoichiometry reaction^8,10^.

If n-heptane is used, the chemical structure of the interfacial compounds becomes R(COOH)_2_.Q_2_ for fumaric and malic acids, respectively R(COOH)_2_.Q_4_ for succinic acid^8,10^. Therefore, according to the above discussions, the increase of the Amberlite LA-2 amount in the organic phase induces the continuous increase of the extraction degrees in the considered experimental range of extractant concentration. Moreover, due to the lower dissociation capacity of succinic acid, this can be more easily solubilized in solvents with lower polarity. Consequently, at low concentrations of aminic extractant (below 5 g/l) the extraction yield of succinic acid is higher than those recorded for the other two acids, the physical process becoming the main way to extracting the solute (Fig. 3).

Basically, Fig. 3 suggests the possibility of selective separation of fumaric acid from fermentation broth depending on the concentration of Amberlite LA-2 in the organic solvent. To confirm this hypothesis, the variation of the selectivity factor, above defined, with the aminic extractant concentration dissolved in the three organic solvents was analysed.

**Figure 4:**
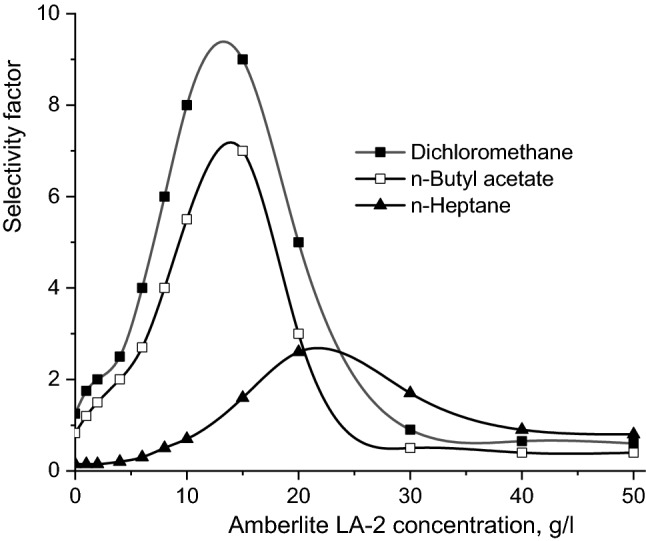
Influence of Amberlite LA-2 concentration on selectivity factor (pH 2, mixing intensity = 0.25 m/s, aqueous phase viscosity = 1 cP).

From Fig. 4 it can be seen that by reducing the solvent polarity the value of the Amberlite LA-2 concentration corresponding to the maximum of the selectivity factor increases from 15 to 20 g/l, simultaneously with the reduction of the maximum values of selectivity. Obviously, this difference is the result of mechanism of interfacial reactions modification, as aforementioned, which requires higher amounts of extractant for a complete reaction with dicarboxylic acids.

The reduction of selectivity factor is the result, on the one hand, of the changes of the reactive extraction mechanism, and on the other hand, of the important increase of the physical co-extraction of the compounds with lower acidity in solvents with lower polarity.

Compared to the variations plotted in Fig. 2, for all studied solvents Fig. 4 indicates that the selectivity factor reaches values considerable higher than 1, which suggests the possibility to control effectively the selectivity by the concentration of the extractant, and less by the pH values of the aqueous phase.

**Figure 5:**
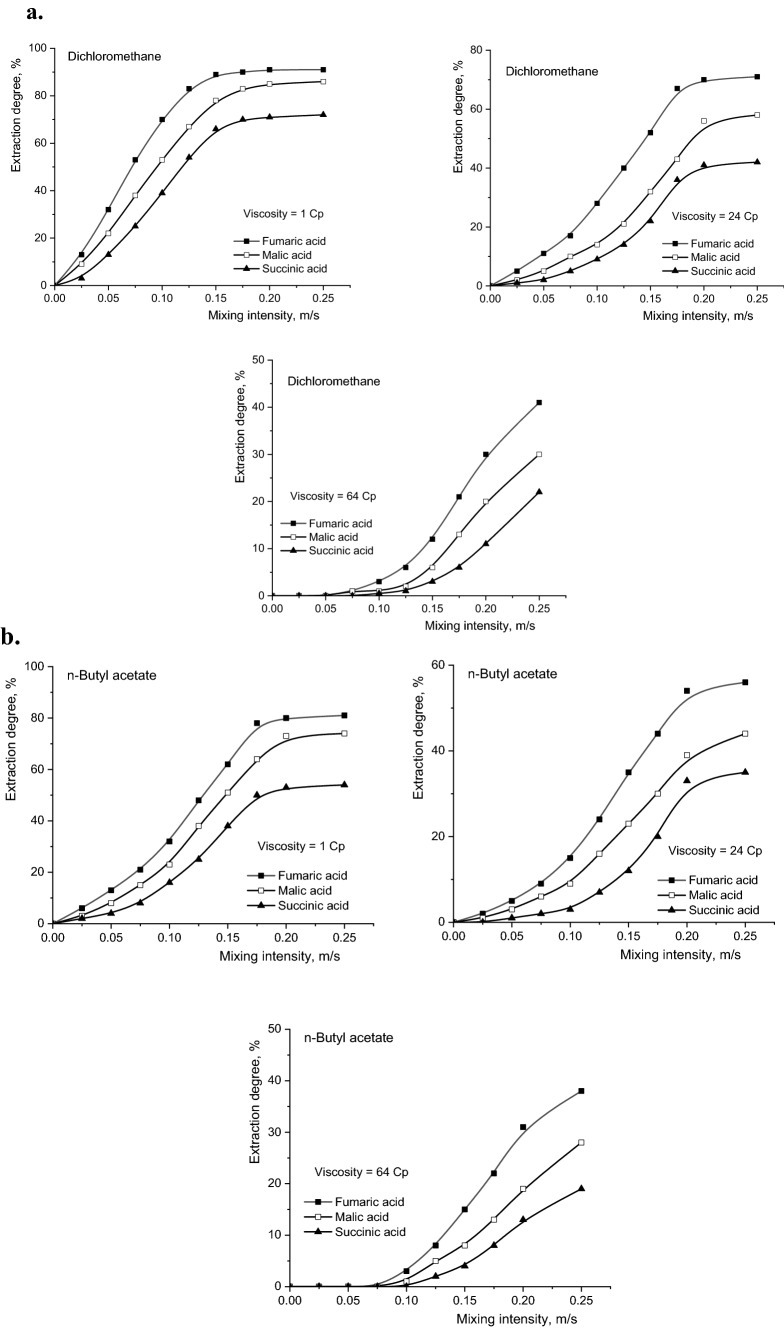

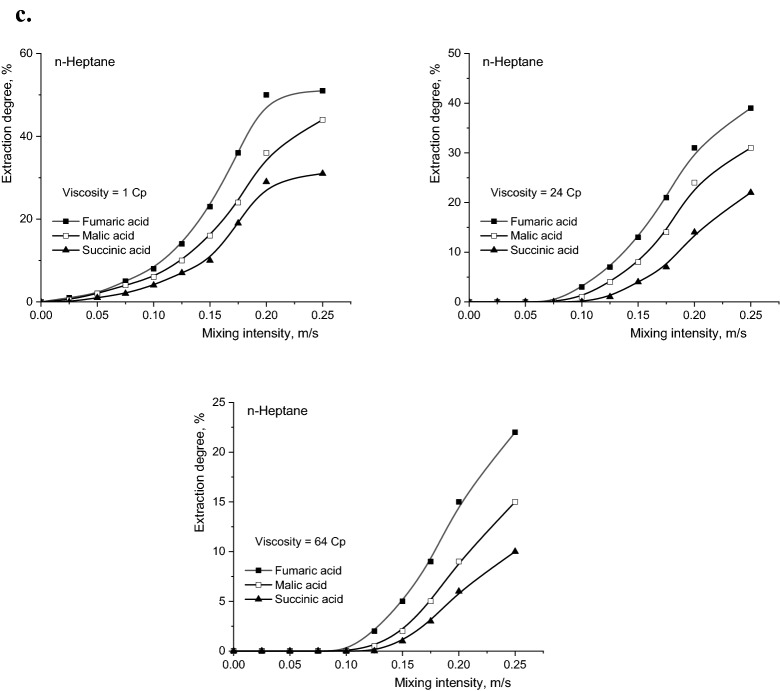
(**a**) Influence of mixing intensity on reactive extraction efficiency of fumaric, malic, and succinic acids for dichloromethane (Amberlite LA-2 concentration = 50 g/l, pH 2). (**b**) Influence of mixing intensity on reactive extraction efficiency of fumaric, malic, and succinic acids for n-butyl acetate (Amberlite LA-2 concentration = 50 g/l, pH 2). (**c**) Influence of mixing intensity on reactive extraction efficiency of fumaric, malic, and succinic acids for n-heptane (Amberlite LA-2 concentration = 50 g/l, pH 2).

The intensity of the aqueous and organic phases mixing represents a factor that could influence the selectivity of the separation, especially in conditions where the molecular masses of the extracted compounds are relatively different and, implicitly, the diffusion rates to and from the reaction region are different. In this context, the influence of mixing intensity of the two phases must be correlated with the viscosity value of the initial aqueous solution. Figure 5a–c show the variations of the degrees of extraction with the mixing intensity, for the three solvents, at different values of the aqueous phase viscosity. The intensity of vibratory mixing was calculated as the product of the vibrations frequency and their amplitude.

The primary analysis of the dependencies plotted in Fig. 5 suggests the existence of two types of limiting steps that control the reactive extraction of the studied acids. The domain of the mixing intensity that corresponds to the continuous increase of the extraction degree is associated with the diffusion regime, in which the main resistance to the total extraction process is the diffusion of the system components to and from the interfacial region. By maintaining the level of the reactive extraction efficiency with intensifying the mixing indicates the change of diffusional regime into the kinetic one, the rate of interfacial chemical reaction becoming the main resistance to this process.

The relative importance of the two types of limiting steps, as well as the extension of the domain of vibrational mixing intensities corresponding to one or other of these resistances, are correlated with the size of the solute molecule and significantly depend on the viscosity of the aqueous phase and the polarity of the organic phase.

From Fig. 5 it can be observed that the general variations recorded for fumaric and succinic acids are similar, but rather different compared to those obtained for malic acid. The explanation is related to very similar molecular sizes of fumaric and succinic acids, both lower than malic acid molecule size.

For systems with higher viscosity, by increasing the mixing intensity the nature of overall extraction process limiting step can be changed, respectively the transition from the diffusion regime to the kinetic one becomes possible (the value of the mixing intensity corresponding to this transition is called critical value). Moreover, the critical value increases with the reduction of dielectric constant of the solvent, due to the decreased solubilisation capacity of the solvents with low polarity and, implicitly, with the amplification of the resistance to the diffusion processes. Thus, by increasing the viscosity from 1 to 24 cP, the critical value of mixing intensity for fumaric and succinic acids increases from 0.15 to 0.175 m/s for dichloromethane, from 0.175 to 0.20 m/s for n-butyl acetate, respectively from 0.20 to over 0.25 m/s for n-heptane. Under the same experimental conditions, the critical value for mixing intensity for malic acid extraction increased from 0.175 to 0.20 m/s for dichloromethane, from 0.20 to over 0.25 m/s for n-butyl acetate, but with any modification of the limiting step nature for n-heptane.

If the viscosity of the aqueous phase increases strongly, becoming 64 cP, the critical value of the mixing intensity cannot be reached in the considered experimental domain for none of the studied acids.

**Figure 6:**
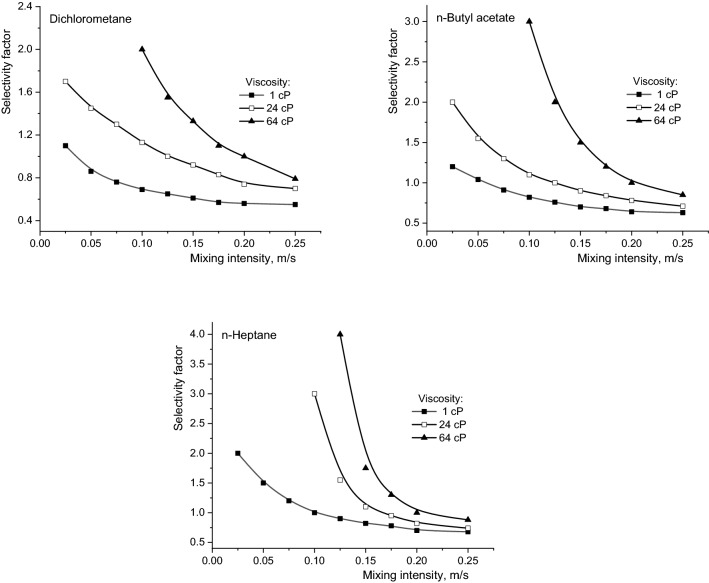
Influence of mixing intensity and viscosity on selectivity factor (Amberlite LA-2 concentration = 50 g/l, pH 2).

Apart from the conclusions regarding the nature of resistances that may appear in the reactive extraction process of carboxylic acids, the increase of the viscosity of the aqueous phase cumulated with the diminution of the organic phase polarity induces the important reduction of the reactive extraction efficiency. Therefore, for the maximum mixing intensity considered in the experiments, by increasing the viscosity in the domain 1–64 cP, the degree of extraction of fumaric acid was reduced by 4.15 times for extraction in n-heptane compared to extraction in dichloromethane. Using identical conditions, the decrease was 5.75 times for malic acid and 7.20 times for succinic acid. The order of the extraction yield decrease is related to the acidities of the extracted compounds. Figure 6 suggests the possibility to reaching high selectivity under less intense mixing by increasing the viscosity of the aqueous phase and by reducing the dielectric constant of the solvent. As it was discussed above, these conditions correspond to the diffusional regime of reactive extraction and are the consequence of the favourable effects of higher acidity and smaller size of the fumaric acid molecule compared to the other two dicarboxylic acids. The intensification of the mixing determines the decrease of the selectivity factor, this phenomenon becoming more pronounced at higher viscosities and solvents lower polarity.

However, due to the low extraction yields recording for the diffusional regime, the mentioned extraction conditions are not practically effective for reaching simultaneously high selectivity and high efficiency of fumaric acid extraction. By combining the pH value and Amberlite LA-2 concentration, the efficient and selective separation of fumaric acid can be achieved. In this regard, selective extraction was performed with 15 g/l Amberlite LA-2, at a pH value of the aqueous solution of 3 and a mixing intensity of 0.175 m/s (Fig. 6).

**Figure 7:**
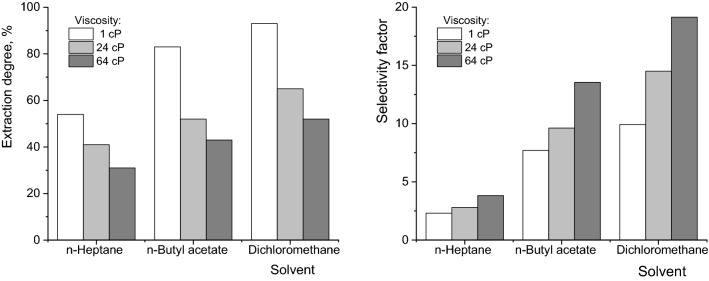
Influence of solvent polarity on extraction efficiency and selectivity factor for fumaric acid.

For the conditions mentioned above, depending on the viscosity of the aqueous phase and the polarity of the solvent, from Fig. 7 it can be seen that the maximum extraction yield of fumaric acid can exceed 90%, this value being associated with a selectivity factor of 10. The maximum value of selectivity factor was 20 and corresponds to an extraction degree of about 60%. For these reasons, it can be stated that, regardless of the extraction system, by a multi-stage extraction process, adjusting in each step the Amberlite LA-2 concentration to that corresponding stoichiometrically to the amount of fumaric acid in the aqueous solution, the entire amount of fumaric acid can be extracted, while malic and succinic acids remain in the raffinate.

## Conclusions

The study on the reactive extraction with Amberlite LA-2 of fumaric, malic, and succinic acid from their mixture obtained by *Rhizopus oryzae* fermentation, using three solvents with different dielectric constants (n-heptane, n-butyl acetate and dichloromethane), indicated the possibility to remove selectively the fumaric acid from the mixture, malic and succinic acids remaining in the raffinate.

The separation selectivity is controlled by extractant concentration in solvent, being positively influenced by increasing the organic phase polarity. The increase of aqueous phase viscosity affects the efficiency of these carboxylic acids extraction, but it improves the selectivity of separation. Therefore, by selecting the proper conditions (Amberlite LA-2 concentration corresponding to the stoichiometric amount needed for reaction only with fumaric acid, mixing intensity level which avoids high diffusion rate of larger molecule acids, namely malic and succinic acids) the maximum extraction yield of fumaric acid exceeds 90% and the selectivity factor becomes close to 20.

Because fumaric acid cannot be completely recovered by a single-stage extraction process, its total separation from the mixture is possible using a multi-stage extraction with the adjustment of aminic extractant concentration in each stage to the stoichiometric value corresponding to the reactions only with this acid. The number of required stages is reduced by increasing the solvent polarity and is increased for viscous aqueous solutions.

## Methods

The experimental studies were performed using an extraction column with vibratory mixing. This equipment, described in detail in previous paper, permits high interfacial area and a quick attainment of the equilibrium state^8^. A performed disk with 45 mm diameter and 20% free section was used for phase mixing and its vibrations had a frequency of 50 s^−1^ and amplitude between 0.5 to 5 mm. The position of the perforated disk was maintained at the initial interface between the aqueous and the organic phases and the extraction time was 1 min. A centrifugal separator was used for broken the resulted emulsion at 5000 rpm.

The aqueous phase of extraction systems was represented by aqueous mixture of dicarboxylic acids similar to that obtained by fumaric fermentation with *Rhizopus oryzae*. Therefore, the composition of the used mixture was: 87% (5 g/l—4.3 × 10^–2^ M) fumaric acid, 9% (0.5 g/l—3.7 × 10^–3^ M) malic acid, and 4% (0.25 g/l—2.1 × 10^–3^ M) succinic acid, respectively^6,7^. In order to respect to viscosity of *Rhizopus oryzae* fermentation broths the aqueous phase contained carboxymethylcellulose sodium salt solution with viscosity varying from 1 cP (pure aqueous solution) to 64 cP^11^. The domain for pH value of initial aqueous solution was 1 to 6, the correction being made with solution of 3% sulfuric acid or 3% sodium hydroxide, depending on the indicated pH-value. The pH-values were determined using a digital pH-meter of Consort C836 type and have been recorded throughout each experiment.

The organic phase of the reactive extraction system composed by solvents with different dielectric constants (Table 1), in which Amberlite LA-2, with concentration between 0 and 50 g/l (0.13 M), was dissolved. The volumetric ratio between the organic and aqueous phases was 1.

**Table 1:** Dielectric constants of the solvents used in experiments^13^.

Solvent	n-Heptane	n-Butyl acetate	Dichloromethane
Dielectric constant	1.90	5.01	9.08

The efficiency of reactive extraction system was determined by dicarboxylic acids extraction degree. For this, the acids concentration in the initial aqueous phase and in the raffinate were measured using the HPLC system (UltiMate 3000 Dionex) with an Acclaim™ OA column (4 mm diameter, 150 mm length, 5 μm porous particle), provided with UV detector at 210 nm^8^. The mobile phase was a solution of 100 nM sodium sulfate, its pH-value being adjusted at 2.65 with methanesulfonic acid. The flow rate of mobile phase was 0.6 ml/min. The analysis has been carried out at 30 °C. For determining the acids concentration into the organic phase, the mass balance has been used. Each experiment has been repeated for three times for identical conditions, the average value of the considered parameters being used.
